# Refined repetitive sequence searches utilizing a fast hash function and cross species information retrievals

**DOI:** 10.1186/1471-2105-6-111

**Published:** 2005-05-03

**Authors:** Jeff Reneker, Chi-Ren Shyu

**Affiliations:** 1 Department of Computer Science, University of Missouri, Columbia, USA; 2 Department of Health Management & Informatics, University of Missouri, Columbia, USA

## Abstract

**Background:**

Searching for small tandem/disperse repetitive DNA sequences streamlines many biomedical research processes. For instance, whole genomic array analysis in yeast has revealed 22 PHO-regulated genes. The promoter regions of all but one of them contain at least one of the two core Pho4p binding sites, CACGTG and CACGTT. In humans, microsatellites play a role in a number of rare neurodegenerative diseases such as spinocerebellar ataxia type 1 (SCA1). SCA1 is a hereditary neurodegenerative disease caused by an expanded CAG repeat in the coding sequence of the gene. In bacterial pathogens, microsatellites are proposed to regulate expression of some virulence factors. For example, bacteria commonly generate intra-strain diversity through phase variation which is strongly associated with virulence determinants. A recent analysis of the complete sequences of the *Helicobacter pylori *strains 26695 and J99 has identified 46 putative phase-variable genes among the two genomes through their association with homopolymeric tracts and dinucleotide repeats. Life scientists are increasingly interested in studying the function of small sequences of DNA. However, current search algorithms often generate thousands of matches – most of which are irrelevant to the researcher.

**Results:**

We present our hash function as well as our search algorithm to locate small sequences of DNA within multiple genomes. Our system applies information retrieval algorithms to discover knowledge of cross-species conservation of repeat sequences. We discuss our incorporation of the Gene Ontology (GO) database into these algorithms. We conduct an exhaustive time analysis of our system for various repetitive sequence lengths. For instance, a search for eight bases of sequence within 3.224 GBases on 49 different chromosomes takes 1.147 seconds on average. To illustrate the relevance of the search results, we conduct a search with and without added annotation terms for the yeast Pho4p binding sites, CACGTG and CACGTT. Also, a cross-species search is presented to illustrate how potential hidden correlations in genomic data can be quickly discerned. The findings in one species are used as a catalyst to discover something new in another species. These experiments also demonstrate that our system performs well while searching multiple genomes – without the main memory constraints present in other systems.

**Conclusion:**

We present a time-efficient algorithm to locate small segments of DNA and concurrently to search the annotation data accompanying the sequence. Genome-wide searches for short sequences often return hundreds of hits. Our experiments show that subsequently searching the annotation data can refine and focus the results for the user. Our algorithms are also space-efficient in terms of main memory requirements. Source code is available upon request.

## Background

Many algorithms have been developed recently that search DNA sequences looking for various types of subsequences [1-10]. All of these algorithms were developed to help life science researchers efficiently and accurately find short segments of DNA within entire genomes. Some search for short tandem nucleotide repeat (STNR) sequences that are commonly found throughout the genomes of higher organisms [1-6]. Others search for variable length tandem repeats (VLTR) and multi-period tandem repeats (MPTR) [8]. Most of them require that the sequence be repeated at least a few times in order to guarantee being detected and all of them require that the repeat be of a certain *minimum length*. Many of the algorithms can identify repeats without a priori knowledge of the repeat pattern. They do this by identifying short segments of DNA, termed *words*, and then manipulating the properties of these words as a process moves down the length of the sequence. A word's properties include, for instance: location, distance to the nearest identical word, and Hamming distance [11] to similar words. Once the process is finished, the results are displayed with accompanying statistics. Each new search must proceed from the beginning of the sequence. However, Ning, Cox, and Mullikin [10] have developed a different type of algorithm called 'Sequence Search and Alignment by Hashing Algorithm', SSAHA, that stores information about the locations of DNA words into a hash table. During a homology search, these locations are sorted and then examined for a series of numbers that are word-length apart. Since the hash map is in main memory, this search can be quite fast. For instance, a search through 2.7 GBases of DNA took only 2.20 seconds per query on average while searching for homology with 177 query sequences (104,755 total bases). However, in this experiment, the system required a minimum homology of 2*k *- 1 bases (where the word size *k *= 10) to guarantee a match. Furthermore, under no circumstances can the SSAHA algorithm find matches for sequence lengths less than *k*. Also, since the hash map is in main memory, scaling becomes a problem for multiple species searches.

In addition to SSAHA, other algorithms employ a static index of the database to speed retrievals. For instance, BLAT [12] is very effective for aligning mRNA and genomic DNA taken from the same species. It uses an index of non-overlapping length *k *DNA words (and their positions in the database) to find short matching sequences that can be extended into longer matches. As with the SSAHA algorithm, BLAT cannot locate hits smaller than length *k*. Also, BLAT excludes from the index *k*-mers that occur too often as well as *k*-mers containing ambiguity codes in order to reduce the number of initial matches to extend. This practice improves performance at the cost of missing some hits.

The FLASH [13] and TEIRESAIS [14] algorithms were designed to manage mismatches between a query sequence and sequences in the database. FLASH uses a strategy where non-contiguous DNA words are concatenated and then indexed. The algorithm generates a very large number of concatenated DNA words from each portion of an original string. TEIRESAIS was built to discover patterns in biological sequences by scanning input sequences to locate the set of all patterns with at least minimal support. Then, it builds larger patterns by matching prefixes and suffixes of patterns in the initial set. However, like the SSAHA algorithm, scaling becomes a problem for FLASH and TEIRESAIS for multiple species searches.

While accuracy and efficiency are important to life science researchers, they undoubtedly would like to do more than just locate their query sequences. For example, suppose a researcher knows the sequence of a transcription factor binding site and wishes to search through several species to see what genes this factor might be controlling. Current algorithms could easily generate tens of thousands of hits but leave the researcher with weeks of additional work in order to locate more specific information. If, however, the researcher also had an idea about possible annotation terms that would accompany specific genes of interest, then they could substantially narrow the search results by simultaneously searching for those terms. The expected results would be all genes that contain both the sequence and the annotation terms. This would add value to the search and help the researcher to understand a biological meaning in the results.

We have developed a search algorithm based on a unique and fast hash function that can search for a query sequence of any length in any number of genomic sequences. Like most other recent search algorithms, our algorithm uses the properties of DNA words, or more specifically their location. We have also incorporated an information retrieval function into our hash structure for fast retrieval of the annotation data that accompanies genomic sequences. This can possibly help facilitate knowledge discovery through cross-species conservation of sequences.

## Results

### Algorithm efficiency and utility

We report three experiments in this paper; the first experiment demonstrates the efficiency of our algorithm while the second and third demonstrate the potentials to the life science community. Our current implementation features a Dual Xeon 2.4 GHz processor with 512 KB cache, 2 GB RAM, and 120 GB EIDE 7200 rpm hard drive.

### Experiment 1: Efficiency study

For our efficiency study, a random number generator was used to select locations from within *Arabidopsis thaliana *chromosome 5, which is over 26,000,000 base pairs. Then a sequence was retrieved from each location. For each sequence length, ten thousand different sequences were obtained and searched. Presently, our database consists of 49 different chromosome sequences from *Arabidopsis thaliana*, *Haemophilus influenzae*, *Helicobacter pylori 26695*, *Helicobacter pylori J99*, *Homo sapien *and *Saccharomyces cerevisiae*. Each chromosome was searched for each query. Since we obtained query sequences from a chromosome within our database, we can expect to find at least one hit per query. The average search times and the average number of hits over all chromosomes for each query length are listed in Table 1. For instance, length 4 queries require approximately 56 seconds to retrieve 15,428,878 hits on average. Length 8 queries require just over one second to retrieve an average of 98,141 hits while length 1024 queries require approximately 4.7 second to retrieve 1 hit on average. The discussion below details an explanation of these results.

### Experiment 2: Refining repetitive sequence searches

Our abstract mentioned that a whole genomic array analysis in yeast had revealed 22 PHO-regulated genes. The promoter regions of all but one of them contain at least one of the two core Pho4p binding sites, CACGTG and CACGTT [15]. Using these sequences, we tried a simple test of the utility of our algorithm. First, the yeast genome was searched for both sequences without adding any annotation terms to find 4027 total hits. Assume that we are interested in learning more about these sites. We can try to focus the search by adding annotation terms. Pho-regulated genes are involved in phosphate metabolism in yeast so the term 'phosphatase' was added to the search which narrowed the results to 51 total hits. We found 10 hits for CACGTG, 20 hits for CACGTT, and 21 hits for AACGTG (the reverse complement of CACGTT). If the promoter region is defined as 1500 base pairs upstream of the start codon, then CACGTG was located in 4 different promoters, CACGTT was in 7 different promoters and AACGTG was in 8 promoters. In other words, 19 genes in the yeast genome contain at least one of the two core Pho4p binding sites in their promoter region and also contain the term 'phosphatase' in their annotation data. This figure is 90.5% (19 / 21) of the results reported in reference [15] and gives a high level of confidence for further investigation of these genes. If the promoter region is extended by 1500 base pairs, then there are 8 more hits – Protein IDs 6321642, 14318551, 6320067, 6324664, 6319971, 6321700, 6322880, and 6325061. These searches were repeated for the other genomes in our database and the results are shown in Table 2.

### Experiment 3: Cross-species study

In this experiment, the findings from a published work involving phase-variable sites in *Helicobacter pylori *were used as an impetus to identify a potentially new phase-variable site in *Haemophilus influenzae*. Furthermore, there is supporting evidence for the new site in another published work involving *Haemophilus influenzae*. Although the experiment presented is specific to phase-variable sites in two species of microorganisms, the methodology is flexible enough to be applied to other similar biological problems.

A recent study [18] of *Helicobacter pylori 26695 *and *J99 *reported 46 candidate phase-variable genes that were identified by either having a homopolymeric tract greater than or equal to (G)_7_, (C)_7_, (A)_9_, and (T)_9 _or having a dinucleotide repeat greater than or equal to four copies. Seven of the 46 genes reported in the study were classified as lipopolysaccharide-biosynthesis related. A search of our database was conducted for the same set of repetitive sequences in the same species. We refined the search by concurrently searching for the term 'lipopolysaccharide' similarly to the previous experiment. The search returned eight distinct genes – two of them (Protein ID 15611217, and 15611264) were identified by having a (CT)_4 _repeat in their open reading frames. This finding was used as an impetus for a new search in a different species.

Assuming that (CT)_4 _also plays a role in the phase-variable properties of lipopolysaccharide genes in other microorganisms, a search for (CT)_4 _was conducted in *Haemophilus influenzae*. Of the 20 total hits, one gene (Protein ID 16273438) is described as a 'Lic-1 operon protein'. This gene is a lipopolysaccharide biosynthesis-related gene because 'Lic-1' is a homologue of lipopolysaccharide and 'operon' is a group of closely linked genes that produces a single messenger RNA molecule in transcription. The paper by Hood et.al. [19] reports three phase-variable lipopolysaccharide biosynthesis genes (Lic1, Lic2, and Lic3) in *Haemophilus influenzae *that were identified by multiple (>4) CAAT repeats. A search for (CAAT)_4 _in *Haemophilus influenzae *with a concurrent search for 'Lic-1' returned Protein ID 16273437 which abuts to Protein ID 16273438 and is part of the same operon. Thus, the 'Lic-1 operon' of *Haemophilus influenzae *contains both (CAAT)_4 _repeats as well as (CT)_4 _repeats – separated by approximately 1400 base pairs.

A molecular biological analysis would be required to verify whether the (CT)_4 _repeat in the Lic-1 operon plays a role in phase-variability, but since it is known in *Helicobacter pylori *[18] it might also in *Haemophilus influenzae*. The purpose of the experiment in this paper is to illustrate how cross-species searches using our database can quickly help discern hidden correlations in the data.

The search algorithms presented here are available on our web site [16] so that users can conduct experiments similar to the ones presented above. Users wanting a brief introduction to the web site can read the paper [17]. The current paper describes the hash function in detail and gives example searches. Here we also present detailed experiments which are not found in the previous paper that demonstrate the usefulness of our approach to the research community.

## Discussion

Our hash function described below processes DNA words that are eight bases in length. Therefore, length 8 queries will retrieve one hash bin in an average of 1.15 seconds. Length 4 queries must retrieve 256 contiguous hash bins because there are 256 possible combinations that make a length 4 query into a length 8 query. For longer queries, although the query lengths double, the search times do not because the recursive search function has been optimized to reduce IO operations. When it has been determined that the query is not present, no further IO operations are performed. The search times presented grow at O(*n*) time, where *n *is the number of chromosomes searched. The chromosomes are searched sequentially so searching a very large number of chromosomes should be possible with our system. After the hits have been retrieved for the current chromosome, its pages are free to be swapped by the operating system.

## Conclusion

We have presented a novel algorithm for homology searches in DNA sequences. Our hash function approach can quickly locate exact matches with a query sequence of any length. Also, our new search engine has several information retrieval features to assist researchers in finding functional homologies across species. Several more data mining features are in development. As more species are added to our database, we anticipate a richer data mining experience for the life science research community.

## Methods

### Definitions

#### Necessary concepts

We define a mapping function *m*(A) = 1, *m*(T) = 2, *m*(G) = 3, and *m*(C) = 4 to map DNA bases into digits. We can convert a DNA word into a number by applying the general positional number system conversion function *f*() to *Q *= {*q*_0_*q*_1_*q*_2_...*q*_|*Q*|-1_}:



where *Q *is a DNA word, *m *is the mapping function, *q *is one base of the word, *r *is the radix (four in our case), and |*Q*| is the length of the word. This conversion differs slightly from the usual number system conversion since zero is not used in order to achieve a one-to-one mapping between a DNA word and the radix 10 number system. If *m*(A) = 0, the function could not distinguish between one A and a string of A's. Table 3 lists twenty sequences and their corresponding radix 10 numbers. One caveat to this conversion in our implementation is that we proceed from left-to-right down the DNA word for coding convenience instead of the usual right-to-left. For instance, the codon ATG is (1 * 4^0^) + (2 * 4^1^) + (3 * 4^2^) = 57.

### Algorithms

#### Obtaining sequence and annotation data

We used sequences and annotation data from NCBI [20]. When available, genomic sequence from a FASTA nucleic acid file (*.fna) and annotation data from a protein table file (*.ptt) were used. Human genomic sequence data from build 34.2 was obtained from the BLAST database in FASTA format [21]. Human annotation data for this build was obtained from two different files. The file named gene.q contained the gene annotation which was listed by GeneID. The file named seq_gene.md listed the beginning and ending locations along a chromosome for each GeneID.

#### Constructing the hash table

Sequences in our database are preprocessed as follows. At each base along the sequence, the algorithm counts up *k *consecutive bases to make a *k*-word. Then, it converts the word into an integer *key *as discussed above. If the sequence has *n *bases, then there are *n *- *k *+ 1 keys in the sequence. The *location *of each key is the number of bases from the beginning of the sequence to the first base of the word that made the key. Thus, the pair <*key*, *location*> describes each base along the length of the sequence. For our application, we add one more descriptive attribute to this pair which is the protein ID (*PID*) of the nearest gene to this location. For human sequences, this ID came from the seq_gene.md file while for other sequences it came from the *.ptt file. A hash table file is used to store *locations *and *PIDs *while a related indexing file stores information about the hash table and is accessed via the *key*.

**Figure 1:**
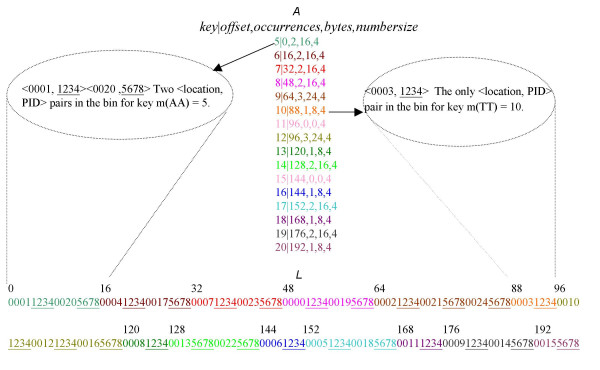
**Index file *A *and data file *L *for sequence *s*_1_**: CAATTACGAGCTCTGCCTACAATGAT. The format for 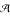 and 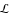 are discussed in the text. To demonstrate how different regions map to different genes, the first 13 bases map to the gene with PID = 1234 and the last 13 bases map to the gene with PID = 5678. We add leading zeroes to each location so that all numbers in 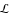 are four bytes and we record this as *numbersize *in each line in 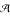. Keys in this example are made from two bases of sequence so there are 4^2 ^= 16 lines in 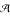 ranging from m(AA) = 5 through m(CC) = 20. Key number m(GT) = 11 and number m(GG) = 15 are not present in the sequence. For clarity, each offset in 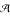 is repeated in the correct position above the line in 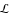 and each PID is underlined. Two arrows map two different lines from 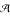 into 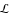 by pointing to two bubbles that show the content of two hash bins.

**Figure 2:**
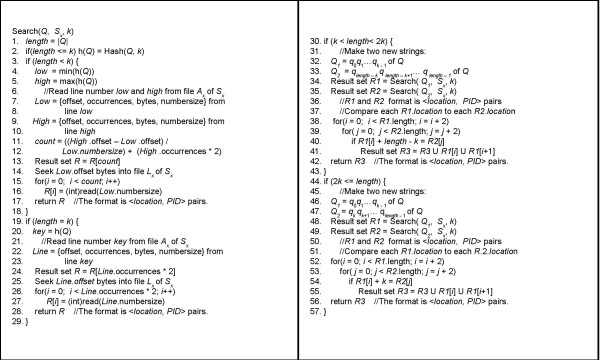
**Database search pseudo code**. The length of the query sequence *Q *determines which block of code will execute. Lines 3 – 18 execute for |*Q*| <*k *(wordsize) while lines 19 – 29 execute for |*Q*| = *k*. If *k *< |*Q*| < 2*k *then *Q *is divided into two *k *length pieces for recursive calls to Search in lines 32 – 35 and the results from these calls are further tested in lines 38 – 41 to obtain the final answer in line 42. If |*Q*| > = 2*k*, a similar block of code is executed in lines 44 – 57. However, a different comparison is made in line 54 as compared to line 40.

Our database contains a set of sequences *S *= {*s*_1_, *s*_2_, ..., *s*_Nd_} where *s*_*i *_is the sequence for the *i*-th chromosome in the database. Each sequence in *S *has two associated files, a list 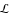 of <*location*, *PID*> pairs and an array 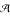 of offsets into 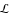 which serves as an index for 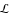. There are 4^*k *^bins in 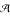, one for each of the 4^*k *^possible *k*-words. After sorting, the algorithm writes each <*location*, *PID*> pair into 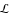 and counts the number of pairs written for each key. This information is written into 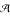 according to the following format:

*key*|*offset*,*occurrences*,*bytes*,*numbersize*

where *key *is discussed above, *offset *is the starting position within 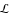 of the first <*location*, *PID*> pair for this key, *occurrences *is the number of <*location*, *PID*> pairs for this key, *bytes *is the number of bytes written to 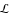 for this key, i.e. the hash bin size, and *numbersize *is the size in bytes of each *location *and *PID*. Figure 1 shows how *offset *plus *bytes *equals *offset *for the next *key*. Each line of 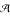 is extended with spaces on the right up to an appropriate number (we use 30) so that all lines have the same length. In this way, when using 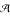 as an index into 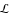, we can easily determine which line to read based on the key. See lines 4–10 and 20–23 of the search algorithm presented in Figure 2. Using larger *k*-words during processing will increase the size of 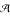 but will not change the size 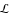. It will, however, decrease the size of each hash bin within 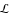.

To distinguish *locations *from *PID*s within 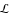, one can either use delimiters between them or add leading zeros to shorter numbers to make all numbers the same size. Using delimiters saves space for shorter sequences while adding leading zeros saves space for longer sequences. For instance, if all PIDs are 8 bytes and all locations are extended to 8 bytes with leading zeros, then this approach begins to save space for sequences longer than approximately 1,000,000 base pairs. The average chromosome length in our database is 65.79 million base pairs. Therefore, our implementation uses leading zeros.

Sequences in FASTA format allow for bases other than A, T, G, and C. For instance, R = (A or G), Y = (T or C), and K = (G or T), etc. However, a radix of 4 will only accommodate A, T, G, and C during the conversion. Therefore, for other bases, the algorithm converts the DNA word into each possible combination. For instance, the word YA is converted into two words, TA and CA, which are both stored into their respective hash bins. Query sequences are similarly converted into each possible combination before searching and these combinations are searched sequentially. Therefore, in sequence *s*_1 _of Figure 1, a user could search for YAA and recover two CAA hits (at positions 0 and 19) and no TAA hits. This approach differs from the SSAHA system which converts all non-standard bases into A's and would not recover any hits since there are no AAA's in *s*_1_. The next two sections show how to use 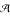 and 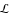 to search for a query sequence within 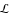.

We also have a table in a relational database to hold gene annotation information. The schema of the Protein table is as follows:

    Protein(species: NUMBER(3), chr: NUMBER(2), begin: NUMBER(8),

       end: NUMBER(8), strand: CHAR(1), length: NUMBER(5),

       PID: NUMBER(10), product: VARCHAR2(3000),

       PRIMARY KEY (PID));

Species is a number that can be de-referenced when needed. The chromosome number, the beginning of the gene, the end of the gene, and the length are self-explanatory. The strand attribute is either + or - depending on the direction of the gene. The PID attribute is the universally unique Protein ID number that allows us to access information on other databases. Finally, the gene product contains a brief description of the function of the gene. Some examples include: Proline /pyrroline-5-carboxylate dehydrogenase, carbonic anhydrase, and lipopolysaccharide biosynthesis protein. Both the PID and the product are indexed based on the frequency of the queries on these attributes.

#### Hash function

The function below takes as input a DNA word *q *and returns the key(s) necessary to locate the correct hash bin(s). It is used by the search function which is presented in the next section. Assume that *k *is still the length of the DNA words that were used to make the hash table in the previous section. If |*q*| <*k*, we would like to expand *q *into a larger sequence domain *q*', where |*q*'| = *k*, while still retaining all of the properties of *q*. One possible way is to add every combination of A, T, G, and C to the left of *q *such that the final length is *k*. Once again, our algorithm works from left to right for coding convenience. Another simpler way is to add A's to the left of *q *to get the word

*q*_*Low *_= A_1_A_2_...A_*k*-|*q*|_*q*

and to add C's to the left of *q *to get the word

*q*_*High *_= C_1_C_2_...C_*k*-|*q*|_*q*

so that |*q*_*Low*_| = |*q*_*High*_| = *k*. By applying the conversion function to *q*_*Low *_and *q*_*High*_, the low and high values of a range of numbers that represents *q *within *q*' are found. Furthermore, the numbers in this range are consecutive. The input to the function, *q*, is the entire query, *Q*, if |*Q*| < = *k *and is a *k*-length subsequence of *Q *if |*Q*| >*k*. The search function presented below determines which case is appropriate.

    Hash(*q*, *k*)

       *offset *= *f*((A)_*k*_)

       if(|*q*| <*k*) //Case 1

          *q*_*Low *_= A_0_A_1_...A_*k*-|*q*|_*q*

          *q*_*High *_= C_0_C_1_...C_*k*-|*q*|_*q*

          h(*q*) = {*f*(*q*_*Low*_) - *offset*, *f*(*q*_*High*_) - *offset*}

       else if(|*q*| = *k*) //Case 2

          h(*q*) = {*f*(*q*) - *offset*}

       return h(*q*)

Case 1 returns two keys that bound 4^*k*-|*q*| ^contiguous hash bins. Case 2 returns only one key for one hash bin. An offset is subtracted from each conversion so that the smallest key, (A)_*k*_, will equal zero, the next larger key will equal one, and so forth.

#### Searching the database

We now present an algorithm called Search to locate all occurrences of a query sequence *Q *within the database (see Figure 2). The database *S *contains a set of sequences {*s*_1_, *s*_2_, ..., *s*_Nd_} where *s *is the sequence of a chromosome. All sequences in *S *are searched separately with their union giving the final result. Each sequence *s*_*x *_has two associated files, the array index  and the list of offsets , which are used differently during a search based on the *length *of *Q*. Since our implementation uses *k *bases of DNA per *k*-word, the three possibilities for *length *are shorter than *k*, equal to *k*, and longer than *k*. In all three cases, the algorithm returns <*location*, *PID*> pairs of all occurrences of *Q*. By taking the reverse complement of *Q *and reapplying the search algorithm, we can search the opposite strand of each sequence.

##### Case 1 – Query length shorter than k bases

There is a set of keys derived by extending *Q *to *k *bases with all possible combinations of sequence and then applying the hash function to each sequence. A range for this set is found by adding A's (or C's) to the left hand side of *Q *until *length *equals *k *and converting to get the low key (or the high key). Since 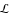 is ordered, this range is continuous from low to high. Next, 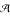 is consulted to determine the *offset*, the *numbersize *in bytes, and the *count *of the numbers to read from 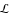. Then, the hard drive's read/write head seeks *offset *bytes into 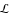 and loads into main memory only the pages necessary for reading. See lines 3–18 of Figure 2.

##### Case 2 – Query length exactly k bases

*Q *is converted into its key and 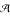 is consulted to find the *offset*, the *numbersize *in bytes, and the *count *of the numbers to read. Then, the read/write head seeks to the correct position within 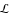 and loads into main memory only the pages necessary for reading. See lines 19–29 of Figure 2.

##### Case 3 – Query length longer than k bases

The search algorithm divides *Q *into two parts, *Q*_1 _and *Q*_2_, where *Q*_1 _is the first *k *bases of *Q *and *Q*_2 _is the last *length *- *k *bases of *Q*. Recursive calls to Search with *Q*_1 _and *Q*_2 _retrieve two sets of results having the format <*location*, *PID*>. They are named *R*_1 _and *R*_2_. After retrieval, we find matches by comparing *locations*. If *length *is greater than *k *and less than 2*k*, then *Q*_1 _and *Q*_2 _will overlap and a match will be a location from *R*_2 _that is (*length *- *k*) greater than a location from *R*_1_. If *length *is greater than or equal to 2*k*, then *Q*_1 _and *Q*_2 _will abut and a match will be a location from *R*_2 _that is *k *greater than a location from *R*_1_. See lines 30–57 of Figure 2.

Retrieval IO is the most time-consuming step in our algorithm. Let *n *be the length of a sequence. If we assume an even distribution of each *k*-word in the sequence then there will be approximately *n */ 4^*k *^*k*-words per hash bin. If each word and each PID are 8 bytes, for instance, then there will be approximately 16*n */ 4^*k *^bytes per hash bin. As an example, with *n *= 200,000,000 bases and *k *= 8 there are 48,828 bytes per hash bin on average. Page sizes usually range from 4,096 bytes up to 4,194,304 bytes. If we assume a page size from the upper half of this range then in this example we can safely estimate for short queries (6 to 16 bases) that the algorithm will only retrieve one or two pages per search. This keeps retrieval times low and allows our system to perform well even with very large hash map files each with 4+ GB made from 200+ million base pair sequences.

#### Examples of searches

As an example of each category of search with *k *= 2, we will search for *Q *of lengths 1, 2, 3, and 4 from *s*_1 _of Figure 1. Line numbers in this section refer to the pseudo code in Figure 2 unless indicated otherwise. Lines 3 through 18 are used to search for "G" within *s*_1_. Hash("G", 2) in line 2 returns the smallest and biggest keys from the set of keys {13 ("AG"), 14 ("TG"), 15 ("GG"), 16 ("CG")}. The key for (A)_2 _from Table 3 is 5. Therefore, in line 4 and 5, index line *low *= 13 - 5 = 8 and index line *high *= 16 - 5 = 11. Line 8 from 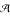 in Figure 1 is 13|120,1,8,4 (the lines begin at zero) and line 11 is 16|144,1,8,4. Applying lines 11 and 12 of the algorithm, we get *count *= ((144 - 120) / 4) + (1 * 2) = 8. Thus, we seek 120 bytes into 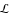 and read 8 numbers which are all 4 bytes long. The result set is [<0008, 1234> <0013, 5678> <0022, 5678> <0006, 1234>] as <*location*, *PID*> pairs. Going back to *s*_1_, all occurrences of "G" have been found (see table 6):

Next, lines 19 through 29 will find exact matches to "TT". The key for "TT" = 10 so the index line from 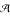 in Figure 1 is 10 - 5 = 5 which is 10|88,1,8,4. In line 25 through 27 of the algorithm, we seek 88 bytes into 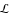 and read 2 numbers which are both 4 bytes long. The result set is [<0003, 1234>] as a <*location*, *PID*> pair. From the original sequence *s*_1 _the only occurrence of "TT" is at location 3.

Now we will illustrate finding "CAA" as a disperse repeat. Disperse repeats are repeats that are not adjacent. From lines 32 and 33 of the search algorithm, *Q*_1 _= "CA" and *Q*_2 _= "AA". Lines 34 and 35 are recursive calls to Search that return *R1 *= [<0000, 1234> <0019, 5678>] and *R2 *= [<0001, 1234> <0020, 5678>] as <*location*, *PID*> pairs. The locations in *R1 *are 0 and 19 and in *R2 *are 1 and 20. Applying lines 36–41, *R1 *[0] + 3 - 2 = *R2 *[0]. Similarly, *R1*[2] + 3 - 2 = R2[2]. So, the pairs [<0000, 1234> <0019, 5678>] are returned as the result set.

Finally, we will illustrate finding the tandem repeat (CT)_2_. Tandem repeats are adjacent repeats. So this example will find "CTCT" as a tandem repeat of "CT". By applying lines 46 and 47 of the algorithm, *Q*_1 _= "CT" and *Q*_2 _= "CT", too. In this example, *R1 *= *R2 *= [<0010, 1234> <0012, 1234> <0016, 5678>]. Applying lines 50–55, *R1 *[0] + 2 = *R2*[2]. This is the only pair of locations that meet the criterion, so the result set is [<0010, 1234>].

#### Information retrieval using query expansion

We conducted the following experiments to examine the best approach for query expansion [11] using the Gene Ontology (GO) database [22]. The first step was to determine the correlation between individual NCBI annotation terms and GO terms. There were 14,349 unique NCBI annotation terms in our database. Of these terms, we found 160 (1.12%) matching terms within the GO database. There were also found 160 inexact matching terms by searching for similarity (i.e. using the LIKE keyword in the WHERE clause instead of equality). Next, we broadened the matching criterion even further by testing unique NCBI terms similarity to GO phrases. The NCBI term was surrounded by wildcard characters (i.e. %term%) and again tested for similarity. This search gave 561,294 matching phrases. We conducted similar experiments with two and three term windows. The results are shown in Table 4. Next, we expanded the GO terms retrieved from the previous experiments by including their unique siblings. Siblings are defined as terms (or phrases) having the same parent in the term2term table in GO. For example, "apoptosis" and "hypersensitive response" are siblings of "autophagic cell death" which share the same parent, "programmed cell death". The results are presented in Table 5. Presently, based on these experiments, we expand annotation terms by querying the GO database with two term windows and including all siblings of GO phrases. This is in an attempt to include other relevant terms in the search.

## Authors' contributions

The hash function algorithms were devised and implemented by JR who also prepared the manuscript. CRS provided essential guidance during development of the algorithms as well as during preparation of the manuscript.

## Figures and Tables

**Table 1:** Time study over 3.224 GBases using randomly obtained sequences

Query Length (Bases)	Average Search Time (Seconds)	Average Number of Hits
4	56.40	15,428,878
8	1.147	98,141
12	2.254	2308
16	2.235	121
32	2.451	1.55
64	2.517	1.15
128	2.648	1.07
256	2.956	1.03
512	3.598	1.01
1024	4.738	1.01

**Table 2:** Cross-species search for PHO4P binding sites, CACGTG and CACGTT (with reverse complement)

	*Arabidopsis thaliana*	*Haemophilus influenzae*	*Helicobacter pylori 26695*	*Helicobacter pylori J99*	*Homo sapien*	*Saccharomyces cerevisiae*
Total Hits In Genome	30,056	1077	81	119	67,881	4027
Hits With 'Phosphatase' Present In Annotation	267	1	0	0	535	51
Percentage Of Hits With 'Phosphatase' Where Hit Is In Promoter (3000 Base Pairs Upstream)	53.9	100	0	0	28.8	52.9

**Table 3:** Conversions between DNA sequences and the radix 10 number system

SEQ	NUMBER	SEQ	NUMBER	SEQUENCE	NUMBER	SEQUENCE	NUMBER
A	1	(C)_3_	84	AATGCT	3,301	GCTCACTG	62,003
C	4	(A)_4_	85	AGTGTCA	8,941	GGGAGGTGAA	388,991
(A)_2_	5	(C)_4_	340	ATGGGGT	12,281	GGGCGGAATT	679,999
(C)_2_	20	(A)_5_	341	GTAGATAA	23,003	GTTTCCTGCG	1,111,211
(A)_3_	21	(C)_5_	1,364	GCGGCTGA	32,003	GCTAAAAGGC	1,299,827

**Table 4:** NCBI terms correlation to gene ontology (GO) terms

	14,349 Unique NCBI One Term Windows	18,934 Unique NCBI Two Term Windows	24,747 Unique NCBI Three Term Windows
GO Term Equality	160	164	73
GO Term Similarity	160	164	73
GO Phrase Similarity	561,294	6840	818

**Table 5:** Gene ontology (GO) term expansion with siblings

	14,349 Unique NCBI One Term Windows	18,934 Unique NCBI Two Term Windows	24,747 Unique NCBI Three Term Windows
GO Term Equality	16,001	9096	4313
GO Term Similarity	16,001	9096	4313
GO Phrase Similarity	19,137,006	228,624	28,556

**Table 6:** 

Location	2-Word
08	AG
13	TG
22	TG
06	CG
